# The Breast Cancer to Bone (B2B) Metastases Research Program: a multi-disciplinary investigation of bone metastases from breast cancer

**DOI:** 10.1186/s12885-015-1528-y

**Published:** 2015-07-10

**Authors:** Nigel T. Brockton, Stephanie J. Gill, Stephanie L. Laborge, Alexander H. G. Paterson, Linda S. Cook, Hans J. Vogel, Carrie S. Shemanko, David A. Hanley, Anthony M. Magliocco, Christine M. Friedenreich

**Affiliations:** 1 Department of Cancer Epidemiology and Prevention Research, CancerControl Alberta, Alberta Health Services, Room 515C, Holy Cross Centre, 2210 2nd St, SW, Calgary, AB T2S 3C3 Canada; 2 Department of Oncology, Cumming School of Medicine, University of Calgary, Calgary, Alberta Canada; 3 Department of Community Health Sciences, Cumming School of Medicine, University of Calgary, Calgary, Alberta Canada; 4 Division of Medical Oncology, Tom Baker Cancer Centre, Cancer Control Alberta, Alberta Health Services, Calgary, Alberta Canada; 5 Division of Epidemiology, Biostatistics and Preventive Medicine, Department of Internal Medicine, University of New Mexico, Albuquerque, New Mexico USA; 6 Department of Biological Sciences, Faculty of Science, University of Calgary, Calgary, Alberta Canada; 7 Department of Medicine, Cumming School of Medicine, University of Calgary, Calgary, Alberta Canada; 8 Department of Pathology, Moffitt Cancer Center, Tampa, FL USA

**Keywords:** Breast cancer, Bone, Metastasis, Cohort, Population-based, Lifestyle, Inflammation, Diet, Physical activity, Vitamin D, Metabolomics, Gene expression, Recurrence, Survival

## Abstract

**Background:**

Bone is the most common site of breast cancer distant metastasis, affecting 50–70 % of patients who develop metastatic disease. Despite decades of informative research, the effective prevention, prediction and treatment of these lesions remains elusive. The Breast Cancer to Bone (B2B) Metastases Research Program consists of a prospective cohort of incident breast cancer patients and four sub-projects that are investigating priority areas in breast cancer bone metastases. These include the impact of lifestyle factors and inflammation on risk of bone metastases, the gene expression features of the primary tumour, the potential role for metabolomics in early detection of bone metastatic disease and the signalling pathways that drive the metastatic lesions in the bone.

**Methods/Design:**

The B2B Research Program is enrolling a prospective cohort of 600 newly diagnosed, incident, stage I-IIIc breast cancer survivors in Alberta, Canada over a five year period. At baseline, pre-treatment/surgery blood samples are collected and detailed epidemiologic data is collected by in-person interview and self-administered questionnaires. Additional self-administered questionnaires and blood samples are completed at specified follow-up intervals (24, 48 and 72 months). Vital status is obtained prior to each follow-up through record linkages with the Alberta Cancer Registry. Recurrences are identified through medical chart abstractions. Each of the four projects applies specific methods and analyses to assess the impact of serum vitamin D and cytokine concentrations, tumour transcript and protein expression, serum metabolomic profiles and *in vitro* cell signalling on breast cancer bone metastases.

**Discussion:**

The B2B Research Program will address key issues in breast cancer bone metastases including the association between lifestyle factors (particularly a comprehensive assessment of vitamin D status) inflammation and bone metastases, the significance or primary tumour gene expression in tissue tropism, the potential of metabolomic profiles for risk assessment and early detection and the signalling pathways controlling the metastatic tumour microenvironment. There is substantial synergy between the four projects and it is hoped that this integrated program of research will advance our understanding of key aspects of bone metastases from breast cancer to improve the prevention, prediction, detection, and treatment of these lesions.

## Background

Breast cancer is the most common cancer in women in North America with over 250,000 cases annually and approximately 45,000 deaths [1, 2]. In patients who develop metastatic disease, 50–70 % will have bone involvement [3–6] and the propensity for primary breast cancer to metastasize to bone has been recognised for over one hundred years since the time of Paget’s speculation on the relative roles of “seed and soil” in the progression of cancer [7, 8]. Approximately 10 % of all breast cancer patients, without evidence of bone metastases at the time of diagnosis, will have a first relapse in bone within five years of their primary diagnosis [3, 9, 10]. Although women with predominant or exclusive bone involvement typically live longer than women with other sites of breast cancer metastasis, these lesions cause serious lingering morbidity as a result of pathologic bone fractures, bone pain, hypercalcemia and spinal cord compression, and eventually culminate in death [6, 11].

Occult micrometastases have been detected in bone stromal aspirates from over 50 % of women at the time of primary breast cancer diagnosis [12–15]. However, there is no current method to identify the features of micrometastases that will eventually progress to create a clinically detectable and symptomatic bone lesion; some may remain dormant indefinitely. Two decades of research have revealed that bone metastasis is a multi-step process of adhesion, invasion, angiogenesis and osteolysis, but the successful prevention, prediction and treatment of these lesions remains elusive. New therapeutic strategies for bone metastases have become available recently [16], however current treatment options are generally palliative.

Bone metastases from breast cancer are predominantly osteolytic although osteosclerotic and mixed lesions can be observed in the same patient [17, 18]. Osteolytic lesions are dominated by osteoclasts that mediate bone resorption during the normal process of bone remodelling [19]. The presence of metastatic breast cancer cells in the bone drives complex interactions between the breast cancer cells, the bone and stromal cells resulting in the recruitment of osteoclast precursors, osteoclast activation and establishment of symptomatic lytic metastases [20–24]. The bone matrix is a reservoir for growth factors that are released during breast cancer induced bone lysis; these growth factors enhance the recruitment and proliferation of osteoclast progenitors and breast cancer cells. This “vicious cycle” involving recruitment of stromal growth factors, activation of osteoclasts, and further proteolysis drives the progressive osteolysis observed in primary breast carcinoma metastasis to bone [25, 26] and is a central target for disruption by current anti-metastatic treatment strategies [27].

The advent of powerful gene profiling technologies has enabled rapid advances in our understanding of the biological basis of bone tropism in subsets of metastatic breast cancer [28–30] and suggested that breast cancer cell recruitment to metastatic sites is attributable to the activation of specific molecular programs in the primary tumour [31–33]. However, despite almost a decade of subsequent research, no primary tumour gene expression signatures have yet been independently validated in humans [34, 35]. Selecting patients at greatest risk of bone metastases, by characterizing features of the primary tumour, could direct the optimal use of therapeutics such as bisphosphonate and receptor activator of nuclear factor-κB ligand (RANKL) inhibitors [36]. The early detection of bone metastases, prior to radiological detection or the onset of skeletal pain, by serum factors or metabolomic profiles, could also potentially direct treatment more judiciously than as a default adjuvant therapy. In addition to the prediction of bone metastases and the selection of patients for therapies, there is some evidence that certain lifestyle factors, particularly vitamin D sufficiency and use of non-steroidal anti-inflammatory drugs (NSAID) use, can influence a patient’s risk for developing metastatic disease following their primary diagnosis, [37–40]. Understanding the potential role and contribution of lifestyle factors to the risk of developing bone metastases would inform optimal lifestyle advice following primary breast cancer diagnosis. Finally, characterising the specific breast cancer cells or molecular signaling conditions that lead to overt metastases could identify potential therapeutic targets for tertiary prevention.

### Program overview

**Fig. 1:**
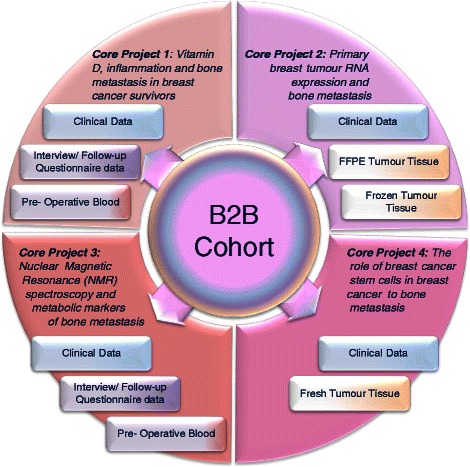
B2B Research Program overview. Clinical data, questionnaire and interview responses, and biospecimens collected from the B2B Cohort are used to support each of the four Core Projects

The Breast Cancer to Bone Metastases (B2B) Research Program is an on-going, dynamic, interdisciplinary research program addressing multiple aspects of breast cancer to bone metastases. Addressing these complex questions is beyond the scope of a single project or investigator. Consequently, we assembled a core research team with expertise ranging from basic science to population health science to clinical care. Four core projects, each investigating an important aspect of breast cancer bone metastases, are based on the biologic samples and data collected from a prospective cohort of breast cancer patients, the B2B Cohort (Fig. 1). The B2B Research Program was established to support four core projects that examine the lifestyle, pathological, and biologic factors associated with these debilitating lesions. The overall B2B Research Program is approved by the provincial research ethics board (Health Research Ethics Board of Alberta, HREBA) and the University of Calgary institutional ethics board (Conjoint Health Research Ethics Board, CHREB).

#### Core Project 1: Vitamin D, inflammation and bone metastasis in breast cancer survivors

There is convincing evidence to support an inverse association between risk of breast cancer and both vitamin D and calcium status (reviewed in [41]). Furthermore, pre-clinical evidence, from animal models, suggests that vitamin D may impede metastases to bone [42, 43]. However, the role of vitamin D in the development and natural history of bone metastases, in humans, has not yet been investigated. There are several plausible mechanisms by which vitamin D may reduce risk or retard development of bone metastases. Vitamin D exhibits pro-differentiation and anti-proliferative properties [44, 45], including the terminal differentiation of osteoclasts [46]. Accumulating evidence implicates sub-optimal vitamin D status in the development of rheumatoid arthritis, diabetes (types 1&2), multiple sclerosis, psoriasis, cardiovascular disease, and cancer (reviewed in [47]). The etiology of these chronic diseases all involve a suspected inflammatory component compatible with the observed immunosuppressive and anti-inflammatory activity of 1,25-dihydroxyvitamin D, the active metabolite of Vitamin D, [48, 49].

The use of NSAID has recently been reported to reduce breast cancer recurrence [50] and improve survival [51] and several of the genes identified in the bone metastatic program, are associated with inflammatory responses [31]. Therefore, chronic inflammation exacerbated by vitamin D inadequacy may potentiate the recruitment of disseminated breast cancer cells to the bone and the initiation of osteolytic metastatic bone lesions.

During summer in North America, up to 90 % of vitamin D is synthesized in the skin by ultraviolet B radiation [UVB], with the remainder from food and supplements. In the winter, especially for those living at latitudes above 42° latitude (e.g., Boston, MA), diet and supplements are the predominant sources of vitamin D. Therefore, both dietary and supplemental intake and sun exposure must be considered when assessing vitamin D status in a Canadian population. An estimated 25–39 % of all Canadians are vitamin D deficient and the prevalence of vitamin D deficiency increases with age [52].

We will also measure 25-hydroxyvitamin D (25-OHD), parathyroid hormone, calcium, creatinine, albumin, and phosphate in serum, at baseline. Serum interleukin-1β (IL-1B), Interleukin-6 (IL-6), interleukin-8 (IL-8), Interleukin-11 (IL-11) and tumour necrosis factor-alpha (TNF-α) will be measured as part of a 10-cytokine multiplex assay. PTHrP expression will be quantified by automated immunohistochemistry (IHC) (HistoRx®) in the primary tumour. This project is approved by both the University of Calgary institutional research ethics boards (CHREB).

#### Core Project 2: Primary breast tumour RNA expression and bone metastasis

Previous studies have proposed primary tumour gene expression patterns which appear to be candidate molecular pathways for migration to and successful growth in the bone marrow [31, 32]. Some markers appear to be particularly important in the process; these include: CXCR4 (chemokine (C-X-C motif receptor 4), SDF1 (stromal cell-derived factor1, also known as CXCL12), CTGF (connective tissue growth factor), FGF5 (fibroblast growth factor 5), MMP1 (matrix metallopeptidase 1), Il-11, PTHrP and osteopontin. These proteins have acknowledged roles in cell recruitment, angiogenesis, bone lysis, adhesion, migration [53–66] and are currently being evaluated as candidate therapeutic targets for the prevention of metastasis. However, despite the promising results in animal models, subsequent attempts to identify a similarly informative signature in humans have failed. It is likely that systemic factors interact with tumour-specific factors to determine risk of bone metastases [34].

This core project will investigate whether the ability for breast cancer to metastasize to bone is an intrinsic characteristic of the primary breast tumour or if systemic factors are essential. Tumour protein marker expression will be evaluated on tissue microarrays (TMAs) and quantified using fluorescence IHC and the HistoRx® AQUAnalysis digital image analysis platform. Compartment specific analysis of protein expression will be accomplished by the use of compartment-specific stains (4′,6-diamidino-2-phenylindole (DAPI) for nuclei, pan-cytokeratin for tumour cells and the tumour cytoplasm, and vimentin for the non-malignant tumour-associated stroma). In addition, RNA will be extracted from micro-dissected tumour-enriched tissues from each tumour and multiplexed target gene expression will be assayed on a Luminex 200 platform using a custom designed Affymetrix QuantiGene® Plex 2.0 assay. Systemic factors will be measured in corresponding serum samples by multiplexed Luminex protein assays. This project is approved by the University of Calgary institutional research ethics board (CHREB).

#### Core Project 3: Metabolic markers of bone metastasis in breast cancer survivors

Metabolism in cancer cells is clearly distinct from that in normal cells. The shift in energy metabolism from mitochondrial oxidative phosphorylation to an enhanced reliance on glycolysis is commonly referred to as the Warburg effect [67]. Other key metabolic pathways are also commonly dysregulated, including the pentose phosphate shunt, the tricarboxylic acid cycle, lipid and phospholipid turnover, choline metabolism, various redox pathways and nucleotide biosynthesis [68]. Metabolic profiles can be exploited through metabolomic approaches as a potentially powerful method for cancer biomarker discovery. The application of metabolic profiling towards various cancers has been reviewed recently [68, 69] and the use of large-scale metabolic analysis is gaining acceptance in multiple clinical settings [70]. To date, metabolic profiling of serum or urine samples has been used, for example, to distinguish between cancerous and benign growth in pancreatic cancer patients [71], for staging patients suffering from colon cancer [72], for studying the effectiveness of bladder cancer treatments [73], and for distinguishing between ER+ and ER- breast cancer tumours [74].

Recently, it has been suggested that ‘omics’ techniques should be capable of predicting when metastasis to bone in breast cancer patients will occur [75]. Indeed one small-scale study already suggests that this type of prediction may be feasible using a metabolomics approach [76]. The B2B Research Program is based on a larger, prospective study with pre-surgical baseline blood collection and serial samples collected during extended follow-up. We aim to derive a metabolic signature to identify patients at highest risk of metastasis to bone, potentially develop a test for early detection of bone metastatic disease, and provide biologic insights into both staging and transcriptional signatures and subtypes within a single prospective cohort. This research brings the prospect of a personalized treatment approach into focus [77].

Our primary analytic platforms for metabolic profiling are proton NMR spectroscopy and gas chromatography time-of-flight mass spectrometry (GC-TOF-MS). These are well-established methods that both provide quantitative results for polar metabolites [68, 69, 71, 74]. The metabolite profiles will subsequently be analyzed using standard chemometric and multivariate statistical methods [78] to determine a signature associated with bone metastases. Serum samples are relatively non-invasive, provide an alternative to more invasive sampling techniques [79, 80] and would be readily available for diagnostic and prognostic studies in normal clinical settings for the prediction and early detection of metastatic disease and treatment response monitoring. This project is approved by the institutional research ethics board (CHREB).

#### Core Project 4: Breast cancer mediated osteoclast differentiation and bone lysis

The detection of breast cancer cells in bone marrow aspirates from breast cancer patients, even those diagnosed at an early stage of disease, suggests that dissemination of cancer cells is an early event in breast cancer [15, 81]. However, only a subset of disseminated breast cancer cells ever develop into overt metastases [82]. Many authors have suggested that only cells with stem-like properties can progress beyond micrometastases [83]. However, it is unclear whether these stem-like properties are intrinsic or acquired at the metastatic site [84, 85]. Recently the importance of epithelial-mesenchymal transition (EMT) and its reversion to an epithelial phenotype for metastatic colonization has been highlighted [86, 87]. There have also been reports of EMT inducing stem-like properties in cancer cells [88, 89] although the link is not necessarily direct [86].

Breast cancer cells communicate with resident osteoclasts and osteoblasts in the bone marrow to establish predominantly osteolytic lesions. The primary treatments of bone metastases are bisphosphonates and RANKL inhibitors (e.g. Denosumab®, monoclonal antibody to RANKL). We will focus on the contribution of RANKL signalling and RANKL-independent osteoclast activation in the context of breast cancer bone metastases. The interactions of cancer cells and cancer stem cells (tumour-initiating cells) with osteoclasts in the initiation and progression of osteolytic lesions and the signaling pathways that control these cells are areas of intense current research [34, 90]. Determining which disseminated tumour cells can initiate overt metastases [82] and identifying the factors that control their interactions are essential to developing effective therapeutic and preventive strategies.

Putative tumour-initiating cells will be enriched from primary breast tumour tissue, cultured and characterized to examine the signalling pathway interactions within the metastatic microenvironment. Specific candidate signalling pathways will be interrogated for their ability to influence lytic osteoclast formation. Co-culture experiments with breast cancer and bone marrow cells will facilitate interrogation of specific signalling pathways [91]. This project is approved by the Conjoint Health Research Ethics Board (CHREB).

Understanding which subset of breast cancer cells had the potential to establish overt metastases and which signalling pathways contribute to the progression of these lesions will support the early detection and risk assessment for metastases and the development of targeted therapeutics to manage or potentially eradicate bone metastases.

## Methods

### Study design

#### Population-based ascertainment

**Fig. 2:**
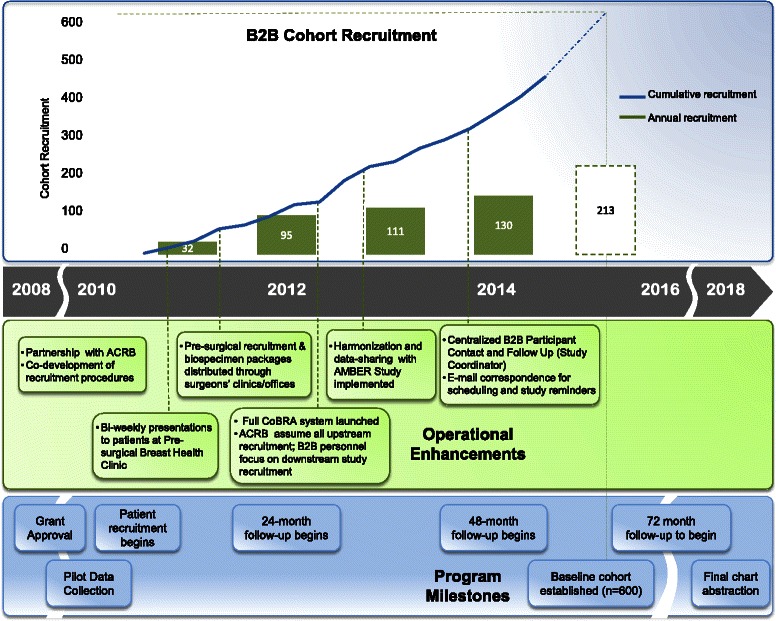
B2B Research Program timeline. Recruitment for the B2B Cohort began in 2010, and steadily increased through successive operational enhancements, key partnerships, and implementation of a centralized biospecimen ascertainment infrastructure

The population-based ascertainment of breast cancer patients for the B2B Research Program was developed in partnership with the Alberta Cancer Research Biobank (ACRB) and the Alberta Cancer Registry (ACR). The ACR is a province-wide cancer registry that has been awarded a Gold Certification from the North American Association of Central Cancer Registries since 1999, indicating the highest quality of completeness, accuracy, and timeliness of cancer reporting. Prior to the establishment of the B2B Research Program, the ACRB focused predominantly on the collection of fresh-frozen tumour tissue from breast cancer patients in addition to a limited collection of largely post-surgical blood samples. The need to recruit a population-based cohort and collect pre-surgical blood samples, led to the development of the Comprehensive Biospecimen Rapid Ascertainment (CoBRA) system (Fig. 2). The CoBRA system is responsible for the ascertainment of patients and their recruitment into the ACRB. The ACRB is approved by both the institutional and provincial research ethics boards (HREBA and CHREB, respectively).

The CoBRA system was designed to identify all newly diagnosed breast cancer patients, within the Calgary area, through seven multiply-redundant mechanisms. The primary mechanism for patient identification is the pathology reports pertaining to their diagnostic (pre-surgical) fine needle and core biopsy. All biopsy reports are submitted to the ACR; a copy of the report is also submitted to dedicated ACRB personnel who are designated as affiliates of the ACR and bound by the code of conduct and training required by all ACR personnel. The additional six supplementary sources of patient ascertainment are outlined in Table 1. Each new patient is recorded in the CoBRA database and all correspondence and patient contacts, pertaining to their informed consent and biologic sample collection for the ACRB, are managed within this system. Potential donors to the ACRB are contacted only after their awareness of their diagnosis has been confirmed. Written informed consent is sought from each patient to donate a pre-surgery (or pre-neoadjuvant therapy, if applicable) blood sample and tumour tissue at surgery if sufficient tissue is available without compromising pathologic assessment or future clinical care. The informed consent process for the ACRB consists of a Consent Information Brochure and a separate consent form on which patients select to participate or not and indicate their willingness to be contacted regarding future research.

**Table 1:** Seven patient ascertainment and recruitment strategies to facilitate comprehensive population-based biospecimen accrual and the potential for differential patient selection associated with each individual approach

Identification method	Description	Potential for selection bias
Alberta Cancer Registry^a^	Pathological evidence of a positive cancer diagnosis provided by the Alberta Cancer Registry.	Cancer registries may not capture 100 % of patient populations and/or may not identify patients with sufficient time for recruitment prior to treatment.
Direct Clinician Referral	Collaborations with key high-volume clinicians including surgeons and oncologists pro-actively introduce the ACRB to patients during pre-treatment consultations	Not all clinicians are supportive or have the time and/or resources to support recruitment initiatives.
Surgical Booking Request	When a patient is diagnosed with a resectable cancer, a surgical booking request is generated to secure a surgery date and surgical suite.	Only includes patients scheduled for surgical treatment for their cancer.
Pre-Admission Clinic	The pre-admission clinic ensures that patients are prepared for a scheduled operation or procedure.	Over-representation of patients with significant co-morbidities and/or are considered at high risk of complications during a medical procedure.
Day Surgery Unit (DSU)	Patients are identified on the operating room slate and encountered in the DSU just prior to their surgery on the day of the operation.	Only includes patients treated for cancer with surgery/excision.
Pre-treatment Patient Education	Numerous programs are available to educate and inform patients prior to treatment.	Patient education sessions are not mandatory; only subsets of broader populations attend these sessions.
Nurse Navigator Referral	Oncology nurses are assigned to patients to help them navigate the continuum of cancer care. They may introduce patients to the ACRB and/or notify the ACRB that a patient has entered their program [113].	Not all nurse navigators are prioritize research recruitment and/or notify the ACRB of patients entering their program.

^a^Additional ethical considerations involving the patient’s awareness of diagnosis must be addressed prior to contacting a patient to obtain informed consent for biobanking

The comprehensive population-based ascertainment of breast cancer patients commenced in February 2010. All potentially eligible participants for the B2B Cohort are selected from patients who agreed to participate in ACRB. The CoBRA procedures will continue to ascertain and recruit patients beyond the time frame of the B2B Research Program, to support additional research projects and biospecimen requests.

#### Study population

**Fig. 3:**
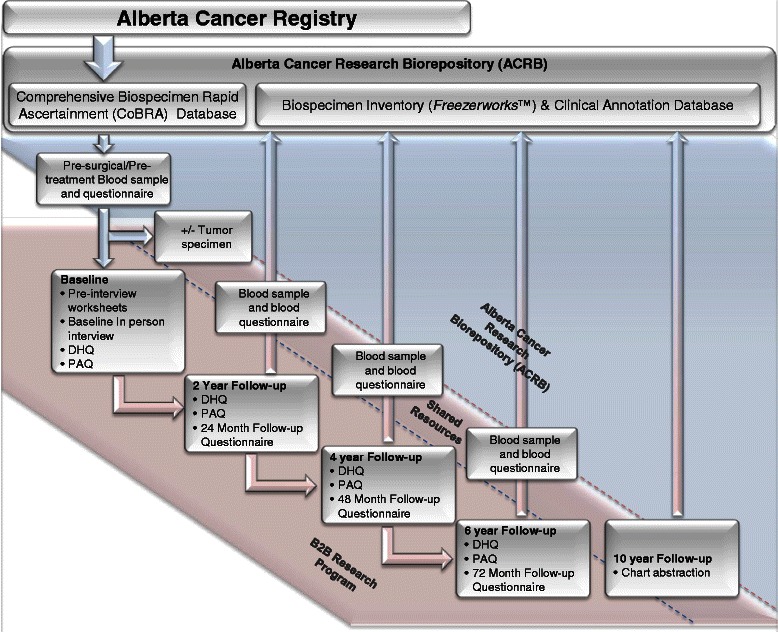
Ascertainment recruitment, data and biospecimen collection and sharing scheme. Newly diagnosed cancer patients are identified through the ACR, and are invited to donate biospecimen samples by the ACRB utilizing the CoBRA infrastructure. Clinical data and biospecimens are stored by the ACRB, and contact information from eligible and consenting participants is sent to study coordinators of relevant research programs. Subsequent blood samples and blood questionnaire data for routine study follow-up are collected by the ACRB and act as a shared resource between the biorepository and research study team

Patients with incident primary breast cancer are eligible for recruitment into the B2B Cohort if they meet the following criteria as determined through the CoBRA database: (1) histologically-confirmed stage I to stage IIIc breast cancer diagnosed between 2010 and 2015; (2) residents of Calgary, Alberta and the surrounding areas; (3) females ≥18 and ≤80 years at initial diagnosis; (4) able to provide informed, written consent and complete questionnaires and an in-person interview in English; and, (5) no previous cancer diagnosis with the exception of cervical in-situ neoplasia (CIN) and non-melanoma skin cancer. Cohort participants must have donated a pre-surgical blood sample to the ACRB (Fig. 3) and indicated willingness to be contacted for future research. The contact details of eligible patients are then exported to the B2B Research Program database for recruitment into the B2B Cohort. The B2B Cohort is restricted to the Calgary area because of the feasibility of processing blood samples collected from community laboratories within 24 h.

#### Recruitment

**Fig. 4:**
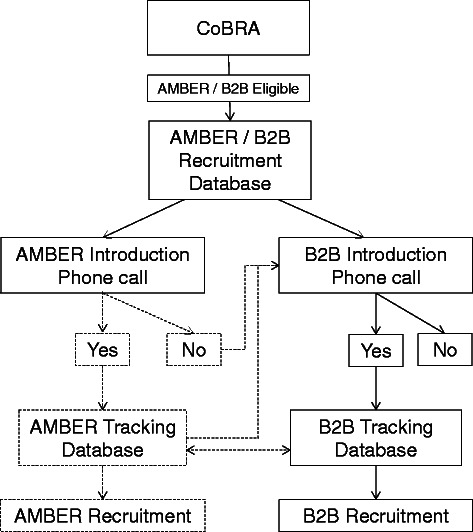
B2B/AMBER participant recruitment. Eligible patients are identified by the ACRB through CoBRA processes, and the contact details of consenting biospecimen donors are sent to a recruitment database shared by both the AMBER and B2B study coordinators. Patients are invited to participate by each study, and if they agree, their information is then imported into the specific study database. Some information sharing occurs between the AMBER and B2B study database, such as whether or not shared questionnaires have been completed

The recruitment of patients into the B2B Cohort was harmonized with recruitment for the **A**lberta **M**oving beyond **B**reast canc**ER** (AMBER) study [92] because the potential participants are drawn from the same population of breast cancer patients (Fig. 4). If a patient agrees to be contacted regarding future research, contact details for all eligible patients are imported into the *AMBER & B2B Recruitment Database*. Patients are first invited to participate in the AMBER study so that their exercise assessments can be completed prior to the delivery of systemic therapy [92].

Patients are invited to participate in the B2B Cohort approximately 6–8 weeks post-surgery, and after potential recruitment into the AMBER study. The B2B Research Program Study Coordinator contacts eligible women by telephone to explain the research program. If the potential participant verbally agrees to receive the recruitment package, their information is imported into the *B2B Tracking Database* and they are mailed a letter of invitation, consent information brochure, consent form and *Pre Interview Worksheets*.

Consenting participants complete baseline worksheets, an in-person interview and post-interview questionnaires within six months of an initial breast cancer diagnosis, with follow-up assessment occurring at 24, 48 and 72-month intervals post-diagnosis. Passive follow-up, through chart abstraction of medical records, will occur at 10 years following the completion of the active follow-up or for those who were lost to follow-up but did not withdraw consent. We anticipate that the recruitment of the baseline B2B Cohort will be completed by August 2015; recruitment of the baseline cohort of >600 participants will have taken a total of 5.5 years. Analysis of data and biospecimens will commence shortly afterwards.

#### Sample size

As a core infrastructure resource, the B2B Cohort sample size was not explicitly based on the power to address a single hypothesis. However, to address our primary, outcome–based hypotheses embedded within the core projects, we will follow all cohort members (~600 patients) for a median of five years. Less than 60 % of breast cancer recurrences are apparent within three years of follow-up but ~80 % are evident after five years of follow-up [3, 9, 10]. Within the 10-year time frame of the B2B Research Program, we expect 70-80 women to present with clinically evident bone metastases [3, 9, 10]. Using serum vitamin D concentrations as an example a specific hypothesis to be tested, broad inter-quintile ranges of serum [25-OHD] that are typical within North American populations (Q1 < 14.9 ng/ml, Q5 > 35.3 ng/ml [93]). Therefore, we anticipate that, for the vitamin D and inflammation analyses, a 20 % difference in vitamin D exposures and inflammatory status between women with and without bone metastases will provide 80 % power to detect a relative risk for bone metastases of 1.8 even with this modest sample size. We anticipate that similar effect magnitudes will be observed in the other core projects.

#### Data collection instruments

##### Pre-interview worksheets

Each participant is mailed pre-interview questionnaires including the *Sun Exposure Worksheets* and *Past Year Dietary Worksheets* as part of their recruitment package. Participants are asked to complete these questionnaires and return them by mail. The *Sun Exposure Worksheets* collect information on residence, work history and vacation history for three time periods: the 12 months prior to breast cancer diagnosis; the calendar year five years prior to breast cancer diagnosis; and the calendar year 10 years prior to breast cancer diagnosis. Although eligible participants must be free of detectable distant metastases at diagnosis, breast cancer cells can be disseminated early in tumour development. Capturing exposure data for an extended pre-diagnostic period, and during follow-up, ensures that exposure can be estimated for the entire period that a patient was at risk of disease dissemination.

The *Past Year Dietary Worksheets* collect data regarding food intake and frequency in the 12 months prior to breast cancer diagnosis, and was developed to assess intake of certain foods and supplements that have high vitamin D and calcium content, consumed at a reasonable frequency by female study participants in Alberta [94]. The sun exposure, dietary, and supplement data will be used to estimate levels of vitamin D during the relevant exposure period.

##### In-person interview

The completed pre-interview questionnaires are scanned using TELEform®, an optical character recognition software program, then verified by study staff for completeness before being exported into the Blaise® computer-assisted interviewing draw 1software program to pre-populate corresponding responses in the *B2B Baseline Interview*. Furthermore, if the participant has completed the AMBER *Baseline Health Questionnaire* (BHQ), those verified responses are also used to pre-populate corresponding responses in the *B2B Baseline Interview*. By pre-populating the interview with information provided by the participants in the pre-interview questionnaires and the AMBER BHQ, we reduce participant interview burden and expedite the in-person interview process.

One of the B2B Interviewers conducts the in-person interview at a time and place convenient for the participant. The *B2B Baseline Interview* is typically an hour in length, and collects information regarding pregnancy and menstruation, menopausal status, hormone replacement therapy, birth control and hormone contraceptive use, personal health history/co-morbidity, medications (over-the-counter and prescription), vitamins, minerals and herbal supplements, mobility and physical activity, sun exposure, diet history, family history of cancer, smoking habits, alcohol consumption history, and demographic information. Following the in-person interview, the participant is provided with a Canadian *Diet History Questionnaire* II (*DHQ II*) and *Past Year Total Physical Activity Questionnaire* (*PAQ*) [95], which is to be completed and returned to the study office by mail. If a B2B participant has already completed the *DHQII* and *PAQ* as part of the AMBER study, these responses are made available to the B2B Research Program to further reduce participant burden.

##### Physical activity questionnaire

The *PAQ* is administered at four time points throughout the study: baseline (post-interview), 24, 48 and 72-month follow-up. The *PAQ* is a self-administered questionnaire in which participants report their occupational, transportation, household and recreational/leisure physical activities over the previous 12 months. Participants report the number of hours spent in each activity per week, allowing for analysis of each individual type of activity as well as a summation of all four categories of activities to determine the participant’s total amount of physical activity over the past year [95]. These measures are expressed as metabolic equivalents for each activity and are reported in total MET-hours/week/year of activity [95].

##### Diet history questionnaire

The *DHQ II* [96] is also administered at four time-points during the study at baseline (post-interview), 24, 48 and 72-month follow-up. It is a self-administered food frequency questionnaire developed initially by the National Institute of Health and then adapted for use in the Canadian population [96]. This FFQ is a comprehensive assessment of dietary intake in the previous 12 months that has 164 questions about 134 food items and includes seasonal intake of a variety of foods, the portion size and frequency of intake for each food item. Responses from the *DHQII* provide comprehensive information on dietary habits, output of nutrients and the amount foods and food groups consumed. Additionally, the dose and frequency of vitamin and mineral supplementation over the past year is also obtained.

##### Biospecimen collection

Each participant’s baseline blood sample is collected as part of their ascertainment and upstream recruitment into the ACRB according to the CoBRA procedures. Participants receive a blood requisition form and are asked to donate a blood sample at any Calgary Laboratory Services location. The baseline collection consists of a 60 ml of blood sample collected in six 6 ml Red Top (clot activator) vacutainers and four 6 ml Lavender Top (EDTA) vacutainers. The vacutainers are transported to a central processing laboratory and fractionated by centrifugation to yield a total of 48 aliquots comprised of 26 serum, 14 plasma, 4 buffy coat and 4 red blood cells (400–500 μl per aliquot) in 1 ml Matrix® 2D-barcoded tubes (Thermo Fisher Scientific Inc.). At the time of blood collection, participants also complete a short *Blood Questionnaire* that records information regarding their fasting status, recent smoking, medication, supplement use, family history of cancer and menstrual status.

Hematoxylin and eosin stained slides corresponding to formalin-fixed paraffin embedded (FFPE) tissue blocks are retrieved for all participants for whom tissue is available. Archived tissue blocks will be requested and retrieved according to the H&E slide pathology review; the pathologist will mark the area of the block from which triplicate 0.6 mm tissue cores should be collected for the construction of TMAs. In addition to the collection of tissue cores, 10 μm tissue scrolls will be collected for the extraction of nucleic acids (DNA and RNA). The RNA will be used for the transcript analysis in sub-project 2 and the DNA will be extracted at a later date for ancillary projects potentially investigating mutational analyses. All blood and tissue samples are stored within the ACRB.

##### Participant follow-up at 24, 48 or 72-months

Additional data and biospecimen collections occur at the specified follow-up intervals of 24, 48 and 72-months from the participant’s primary breast cancer diagnosis. Each month, the Study Coordinator queries the *B2B Recruitment Database* to generate a list of participants eligible for follow-up. The Study Coordinator contacts each participant by telephone to confirm their address and willingness to continue their participation in the B2B Research Program. If they agree, participants are sent a follow-up package that includes a *PAQ*, *DHQ II* and the appropriate *Follow-Up Questionnaire* (24, 48 or 72-month); these are self-administered questionnaires to be completed by the participants and returned by mail to the study office. The *24, 48 or 72-month Follow-Up Questionnaires* request information on: personal health history, breast cancer progression (only at 24 month follow up only), recurrence, contra-laterality and new primary diagnosis, medications (prescription and over the counter), smoking habits, alcohol consumption, sun exposure, dietary intake, mobility and physical activity and anthropometric measurements.

Follow-up blood samples are also collected at 24, 48 and 72 months. Each Follow-Up Package contains a *Research Blood Requisition* form and participants are asked to donate a blood sample at any Calgary Lab Services location. The follow-up blood collections consist of a 30 ml of blood sample collected using three 6 ml Red Top (clot activator) vacutainers and two EDTA vacutainers. Again, the vacutainers are transported to a central processing laboratory, fractionated by centrifugation to yield serum, plasma red blood cells and buffy coat and stored in 32 aliquots of 400–500 μl in 1 ml Matrix® 2D-barcoded tubes (Table 1).

##### Vital status check

The vital status of each participant is checked before each follow-up contact at 24, 48 and 72 months through a record linkage done by the Department of Cancer Surveillance (Alberta Health Services). Vital Statistics Alberta (VSA) provides information on all deaths that occurred in the province to the ACR, on request, with underlying cause of death provided by Statistics Canada to VSA. There is an average three-month time lag between the actual death occurrence and reporting to the ACR. Several mechanisms, such as reciprocal agreements between other provinces and record linkages with the Canadian Mortality Database, exist to capture the deaths of participants who left the province of Alberta after their diagnosis. These agreements and processes ensure that vital status can be determined for over 95 % of participants. Cause and date of death will also be obtained from this source.

##### Medical record abstraction

Medical record abstraction will occur in the final year of the B2B Program operation (commencing August 2018). Health Record Technicians from the ACR will use direct data entry to a medical record abstraction form to collect data from the medical records (both paper and electronic charts) for all participants in the B2B Cohort. The medical record abstraction form was developed from standardized forms used in our past physical activity and breast cancer cohort study conducted in Alberta [97, 98].

Baseline pathologic data, including clinical stage and pathologic stage (according to American Joint Committee on Cancer criteria [99], tumor size, grade, histology, estrogen receptor status, progesterone receptor status human epidermal growth factor receptor 2 status, type and results of computerized tomography or positron emission tomography scans, status of margins (with breast conserving surgery), and pathology of lymph nodes (if surgically sampled) are provided by the ACRB/CoBRA database and verified during the medical record abstraction. Abstracted variables will include the type of surgery, and all treatment and follow-up care including data on chemotherapy, radiation therapy, and hormone therapy. Treatment completion rates will be estimated for chemotherapy and hormone therapy but not for radiation therapy since few patients fail to complete radiation therapy. For chemotherapy completion rate, we will estimate the average relative dose intensity (RDI) received for the originally planned regimen based on standard formulae as we have done in a previous RCT [100]. For hormone therapy, the follow-up questionnaires ask participants to report if they have stopped taking their prescribed hormone therapy at any time before its intended completion and the reasons for stopping.

Disease endpoints are defined according to the Standardized Definitions for Efficacy End Points in Adjuvant Breast Cancer Trials [101]. Our primary endpoint of interest is bone metastasis or a skeletal-related event according to the definition in the NSABP 34 trial (http://clinicaltrials.gov/show/NCT00009945). We will also examine other composite disease endpoints as secondary endpoints including overall survival, distant disease-free survival, distant relapse-free survival, and distant recurrence-free interval. Finally, we will examine the single disease endpoints of death from breast cancer and death from non-breast cancer. For participants who have left the province and who are not known to be deceased, the date of leaving Alberta will be used as the censoring time.

## Discussion

Bone metastases are the most common site of breast cancer metastasis and there are currently no curative treatments available. Consequently, predicting the risk of bone metastasis, identifying modifiable lifestyle strategies to reduce those risks, developing methods for early detection and understanding the fundamental biology and potential therapeutic targets are of the highest priority. The B2B Research Program seeks to address each of these priorities via an integrated program of research based on the population-based prospective B2B Cohort. Each of the core projects (Fig. 1) focuses primarily on one of these priorities; however, the integrated program design and shared data and biospecimen resources enables significant synergy between the projects and potential for additional future hypothesis generation and testing.

A population-based prospective cohort of incident breast cancer patients offers the ideal study design to address the priorities that we have identified. Framing such research within existing randomised controlled trials of treatment would be limited by the availability of biospecimens and likely lack the external validity afforded by the population-based ascertainment [102, 103]. Also, large disease-free prospective cohorts, such as the Canadian Partnership for Tomorrow Project [104], could not deliver the necessary number of outcomes within a reasonable period and are not currently configured to collect epidemiologic data and biologic samples specifically during the crucial peri-diagnostic period or conduct disease-specific follow-up. Although previous studies have conducted population-based recruitment of cancer patients in Alberta [94, 105], there was no existing mechanism to comprehensively identify, contact, obtain consent and track breast cancer patients. Furthermore, the increasingly stringent privacy requirements demanded the development of a system that could accomplish the recruitment and biospecimen collection targets whilst being sensitive to the circumstances of the patients and complying with all relevant privacy regulations. The establishment of the B2B Research Program, the co-development of the CoBRA database and procedures and the partnership with the ACRB all contributed to the creation of the infrastructure to facilitate the current and future population-based prospective recruitment.

The collection of biospecimens is a critical component of the B2B Research Program that required the development of new procedures and the implementation of new technology. Adopting the 1 ml Matrix® 2D-barcoded tubes (Thermo Fisher Scientific Inc.) and the 2D barcode scanner (Thermo Fisher Scientific Inc.) enabled a large number of relatively low-volume aliquots to be collected and tracked for efficient inventory management while avoiding excessive manual labelling and minimizing potential for human error. Two core projects within the B2B Research Program are using serum samples to investigate metabolomic and cytokine profiles associated with the risk for bone metastases. Little has been published on the impact of surgery [106–110] or systemic therapy [111, 112] on serum biomarkers, but the existing literature clearly demonstrates that there are significant changes in blood-based markers in response to both surgery and systemic therapy [106–110]. Consequently, the collection of a pre-surgical blood sample is an eligibility requirement for the B2B Cohort.

The detailed epidemiologic data collected on each participant are collected using several data collection instruments that have been adopted from previously published research studies. The *Pre-Interview Worksheets* comprise two short questionnaires developed for the **OV**arian cancer in **AL**berta (OVAL) Study [94] to improve the assessment of overall vitamin D exposure as well as dietary calcium intake. The computer-assisted baseline *In-Person interview (Blaise®)* was adapted from the interviews created for the Alberta Endometrial Cancer Case-Control Study [105] with modifications to address specific questions related to bone health and inflammation. The PAQ that we use was developed to measure total physical activity in the previous year and has been tested for reliability and validity [95]. The DHQ has also been specifically modified to capture the food items available and consumed in Canada [96]. By using existing instruments or modifying those that had previously been used in similar settings, we have minimized the development costs, taken advantage of existing reliability measures and maximized the comparability of our data with existing and future studies using those instruments.

The B2B Research Program will address several critical aspects of breast cancer bone metastases including prediction, prevention, detection, and biology. Sub-clinical vitamin D deficiency may be a prevalent, yet modifiable, risk factor for breast cancer bone metastases. Optimal vitamin D status may help prevent bone metastasis by a fairly straightforward intervention through its reported ability to attenuate inflammation and proliferation, while promoting apoptosis and differentiation. The transcriptional features of the primary tumour and systemic response could provide a method to determine a patient’s risk of bone metastases at diagnosis to direct appropriate therapies or surveillance. Serum metabolomics offers the potential for early detection of bone metastases. Furthermore, by combining these data with the epidemiologic and clinical data, the impact of modifiable lifestyle factors on metabolites associated with bone metastases might be determined. Finally, the *in vitro* and *in vivo* research can dissect the biologic mechanisms and identify potential therapeutic targets.

### Lessons and limitations

Lessons learned during the set-up and early operation of the B2B Research Program have greatly enhanced the ascertainment and recruit of breast cancer patients in Alberta, through the development of the CoBRA system and refinement of study-specific processes. Translating the comprehensive patient ascertainment into adequate recruitment, and ultimately biospecimen collections, was a significant challenge. However, continual development of these processes and the supporting database has resulted in sustainable recruitment, and biospecimen collection, from ~75 % of breast cancer patients in the Calgary, Alberta area.

We originally proposed a tiered sampling mechanism to enrich the B2B Cohort for stage III cancers who are most likely to develop bone metastases. However, our initial rate of recruitment was insufficient to implement this strategy. The most significant obstacle was achieving patient contact with sufficient time remaining to obtain a pre-surgical blood sample. We introduced several mechanisms to facilitate timely contact with patients during clinic visits, through clinical care-related presentations and during the peri-operative period whilst maintaining the original correspondence-based procedures (Fig. 2); these process enhancements improved our recruitment rates and we anticipate the full baseline cohort recruitment to be completed by August 2015. In addition, we have extended the follow-up program over a longer duration (six years of follow-up instead of the three year period originally proposed) to off-set the lower event rates in early stage patients, to capture a greater number of events overall and maximize statistical power.

In 2013 we obtained approval from our institutional ethics board to introduce email as a method of correspondence between B2B Cohort members and our research team. The pre-interview worksheet package has a B2B Research Program email address with the B2B Research Program Study Coordinator contact details. B2B Cohort members are invited to correspond with B2B Program personnel through this email address and are asked to provide permission for research personnel to contact them through their personal email address. E-mail correspondence has been very popular and facilitates immediate engagement of new participants. It also provides a mechanism to acknowledge receipt of study materials, notify participants of their interviewer’s contact details and to thank participants for their contributions. The use of e-mail has resulted in much more efficient communication with participants with less time spent telephoning, greater accessibility for both participants and interviewers, and a greater number of participants completing the interview portion of the study.

In summary, the B2B Research Program is establishing a population-based prospective cohort of breast cancer patients in which we will conduct four initial core research projects that will address key aspects of bone metastasis in breast cancer survivors. The collection of pre-surgical blood samples and detailed epidemiologic data, at baseline and follow-up, will provide a unique and rich resource to address current and future research. To our knowledge, no equivalent resources are currently available. The biospecimen and data resources established by the B2B Research Program will also enable currently unanticipated important research questions to be addressed in a timely manner. The ultimate goal of this research is to improve the prediction of bone metastasis risk, identify modifiable lifestyle strategies to reduce those risks, and improve our understanding of the fundamental biology and potential therapeutic targets to reduce bone metastases in breast cancer survivors.

## Abbreviations

25-OHD: 25-hydroxyvitamin D
ACR: Alberta Cancer Registry
ACRB: Alberta Cancer Research Biobank
AMBER: Alberta Moving beyond Breast cancER
B2B: Breast Cancer to Bone Metastasis
BHQ: Baseline health questionnaire
CIN: Cervical in-situ neoplasia
CHREB: Conjoint health research ethics board
CoBRA: Comprehensive Biospecimen Rapid Ascertainment
CTGF: Connective tissue growth factor
CXCR4: Chemokine (C-X-C motif) receptor 4
DAPI: 4′, 6-diamidino-2-phenylindole
DHQ II: Diet history questionnaire II
DHQ: Diet history questionnaire
DNA: Deoxyribonucleic acid
DSU: Day Surgery Unit
EDTA: Ethylenediaminetetraacetic acid
EMT: Epithelial-mesenchymal transition
FGF5: Fibroblast growth factor 5
HREBA: Health Research Ethics Board of Alberta
IHC: Immunohistochemistry
IL-11: Interleukin-11
IL-1B: Interleukin-1β
IL-6: Interleukin-6
IL-8: Interleukin-8
MMP1: Matrix metallopeptidase 1
NMR: Nuclear magnetic resonance
NSAID: Non-steroidal anti-inflammatory drugs
PAQ: Physical activity questionnaire
PTHrP: Parathyroid hormone-related protein
RANKL: Receptor activator of nuclear factor-κB ligand
RDI: Relative dose intensity
RNA: Ribonucleic acid
SDF1: Stromal cell-derived factor1
TMAs: Tissue microarrays
TNF-α: Tumour necrosis factor-alpha
TNM: Tumour Node Metastasis
UVB: Ultraviolet B
VSA: Vital Statistics Alberta
